# SurfON trial: study protocol for a multicentre, open-label, randomised controlled trial of early surfactant therapy versus expectant management in late preterm and early term infants with respiratory distress

**DOI:** 10.1186/s13063-025-09271-1

**Published:** 2025-12-01

**Authors:** Elaine M. Boyle, Vasha Bari, Peter J. Bradley, Marie Hubbard, Edmund Juszczak, Benjamin Stenson, David G. Sweet, Ursula Bowler, Christina Cole, Pollyanna Hardy, Andrew King, Louise Linsell, Indrani Manoharan, David Murray, Heather M. O’Connor, Oliver Rivero-Arias, Charles Roehr, Kayleigh Stanbury, Richard Welsh, Joy Wiles

**Affiliations:** 1 Department of Population Health Sciences, University of Leicester, Leicester, LE1 7RH UK; 2 Neonatal Service, University Hospitals of Leicester NHS Trust, Leicester, LE5 4PW UK; 3 National Perinatal Epidemiology Unit Clinical Trials Unit, Nuffield Department of Population Health, University of Oxford, Old Road Campus, Oxford, UK; 4 Bliss, 1 st Floor North, 10–18 Union Street, London, SE1 1SZ UK; 5 Nottingham Clinical Trials Unit, School of Medicine, University of Nottingham, Nottingham, NG7 2RD UK; 6 NHS Lothian, Edinburgh, EH1 3EG UK; 7 College of Medicine and Veterinary Medicine, The University of Edinburgh, 7 Little France Crescent, Edinburgh, EH16 4TJ UK; 8 Regional Neonatal Unit, Royal Maternity Hospital, Belfast, BT12 6BB UK

**Keywords:** Late preterm birth, Early term birth, Respiratory distress, Surfactant therapy

## Abstract

**Background:**

Late preterm and early term infants are a large proportion of all births. Historically, outcomes were thought to be similar to those of full term infants, but recent evidence demonstrates worse outcomes. Although serious morbidity is less common than in very preterm infants, the impact of modest illness in large numbers of more mature neonates is significant, with 30–40% requiring neonatal unit (NNU) admission, primarily for respiratory disease. The SurfON trial aims to evaluate whether early surfactant therapy improves outcomes compared with expectant management in late preterm and early term infants with respiratory distress. The primary objectives are to compare neonatal hospital stay and incidence of severe respiratory failure, with secondary objectives assessing other perinatal outcomes and cost-effectiveness.

**Methods:**

The SurfON trial is a multicentre, open-label, randomised controlled trial involving 45 UK NNUs, aiming to recruit 1522 infants over 30 months. Eligible infants are those born at 34^+0^ to 38^+6^ weeks’ gestation who require noninvasive respiratory support for respiratory distress within 24 h of birth. Infants are randomised in a 1:1 allocation ratio to either early surfactant therapy or expectant management. Infants randomised to the early surfactant therapy group will receive a single dose of CUROSURF^®^, a natural animal-derived surfactant. IMP administration reflects local site policy and procedure and may be completed by any clinician for whom this falls within their usual scope of practice, following confirmation of eligibility. The IMP is a commonly used drug in neonates and is being used in line with standard care. Side effects are uncommon and usually transient. Timing and dosage of IMP administration will be collected. Surfactant is a stock drug for all NNUs and will be administered and accounted for as per local practice.

**Discussion:**

Evidence-based guidance for the management of late preterm and early term infants is sparse, and no previous large randomised controlled trials have focused on this population. Management of respiratory distress in late preterm and early term infants varies. Some clinicians prefer early surfactant administration to prevent deterioration—78others delay treatment to avoid mechanical ventilation. This inconsistency highlights the need for trials to establish an evidence base. Unexpected neonatal morbidity and prolonged hospitalisation cause significant psychological distress for families, impacting breastfeeding and maternal well-being. High intensive care workload and associated costs pose challenges for the NHS (National Health Service). Evidence-based management could improve outcomes and reduce healthcare burdens.

**Trial registration:**

ISRCTN 15915394. Registered on 20th February 2020 https://www.isrctn.com/ISRCTN15915394.

## Administrative information

**Table:** 

Title (1)	SurfON: multicentre, open-label, randomised controlled trial of early surfactant therapy versus expectant management in late preterm and early term infants with respiratory distress
Trial registration {2a and 2b}	ISRCTN: 15915394 — Registered 20th February 2020 https://www.isrctn.com/ISRCTN15915394
Protocol version {3}	SurfON Protocol Version 7.0 25th April 2025
Funding {4}	NIHR Health Technology Assessment (HTA) Programme
Author details {5a}	Elaine M. Boyle — MBChB, MSc, MD, PhD, Professor in Neonatal Medicine and Consultant Neonatologist — University of Leicester, University Hospitals, Leicester NHS Trust Vasha Bari, BSc (Hons) — Trial Manager, National Perinatal Epidemiology Unit Clinical Trials Unit (NPEU CTU), University of Oxford Peter J. Bradley, MA — Director of Services, Bliss Marie Hubbard BA (Hons), MSc, RGN, RNCB, Lead Research Nurse — University Hospitals, Leicester NHS Trust Edmund Juszczak — BSc, MSc, CSci, PhD, Professor of Clinical Trial and Statistics in Medicine, University of Nottingham Benjamin Stenson — MB ChB, MD, FRCPE, FRCPCH, Honorary Professor, and Consultant Neonatologist, NHS Lothian David G. Sweet, MB BCh — Consultant Neonatologist, Belfast Health and Social Care Trust Ursula Bowler, Former Senior Trials Manager, National Perinatal Epidemiology Unit Clinical Trials Unit (NPEU CTU), University of Oxford (Retired) Christina Cole, BSc, MSc, Senior Trials Manager, National Perinatal Epidemiology Unit Clinical Trials Unit (NPEU CTU), University of Oxford Pollyanna Hardy, BSc, MSc, Clinical Trialist Director of National Perinatal Epidemiology Unit Clinical Trials Unit (NPEU CTU), University of Oxford Andrew King, BA (Hons) — Head of Trials Programming, National Perinatal Epidemiology Unit Clinical Trials Unit (NPEU CTU), University of Oxford Louise Linsell, BSc (Hons), MSc, DPhil, Associate Professor, National Perinatal Epidemiology Unit Clinical Trials Unit (NPEU CTU), University of Oxford Indrani Manoharan, BSc, MSc, PhD, Senior Programme Manager/Trial Manager, I Consultancy Ltd., London, UK David Murray, BSc, Senior Trials Programmer, National Perinatal Epidemiology Unit Clinical Trials Unit (NPEU CTU), University of Oxford Heather M. O’Connor — BSc (Hons), MSc, Senior Statistician, National Perinatal Epidemiology Unit Clinical Trials Unit (NPEU CTU), University of Oxford Oliver Rivero-Arias, DPhil — Associate Professor of Health Economics and Senior Health Economist, National Perinatal Epidemiology Unit Clinical Trials Unit (NPEU CTU), University of Oxford Charles Roehr, MD, PhD, Professor of Neonatal and Perinatal Medicine, Bristol Medical School (THS), University of Bristol; Clinical Advisor — National Perinatal Epidemiology Unit Clinical Trials Unit (NPEU CTU), University of Oxford Kayleigh Stanbury, BSc (Hons) — Head of Operations, National Perinatal Epidemiology Unit Clinical Trials Unit (NPEU CTU), University of Oxford Richard Welsh, BEng (Hons) — Senior Software Developer, National Perinatal Epidemiology Unit Clinical Trials Unit (NPEU CTU), University of Oxford Joy Wiles — Quality Assurance Manager, National Perinatal Epidemiology Unit Clinical Trials Unit (NPEU CTU), University of Oxford
Name and contact information for the trial sponsor {5b}	University of Leicester, Research Governance Office Research & Enterprise Division Leicester General Hospital, Gwendolen Road, Leicester LE5 4PW Email: RGOsponsor@leicester.ac.uk Phone: 0116 258 4099/4867
Role of sponsor {5c}	The University of Leicester, as the sponsor of the SurfON trial, holds a central role in ensuring successful execution and oversight of the study. Their responsibilities include taking overall legal responsibility for the management of the research study, ensuring that it is undertaken according to all regulatory requirements, and reviewing all documents prior to ethical submission The sponsor will participate in the interpretation of trial data and the results

## Introduction

### Background and rationale {6a}

SurfON is a multicentre, open-label, randomised controlled trial of early surfactant therapy versus expectant management in late preterm and early term infants with respiratory distress. Around a third of all live births in England and Wales occur late preterm or early term (34^+0^–36^+6^ or 37^+0^–38^+6^ weeks of gestation, respectively). However, respiratory outcomes in this population remain a subject of ongoing debate and clinical uncertainty. While historically considered similar to full term infants, recent evidence suggests that these infants are at increased risk of both neonatal and long-term respiratory complications [1–9].

Respiratory disease is the most common reason for late preterm and early term infants to require neonatal unit (NNU) admission [10–12].

Respiratory distress syndrome (RDS) is a consequence of lung immaturity and deficiency of surfactant, a substance that reduces alveolar surface tension and improves lung compliance. The risk of RDS increases with decreasing gestation; however, although respiratory maturity is often assumed, it also occurs in late preterm and early term infants [13, 14]. Other common neonatal respiratory conditions cause secondary deficiency of surfactant production [14, 15]. While the benefits of early surfactant therapy have been established for very preterm infants (< 32-week gestation) [16, 17], its role in late preterm and early term infants with respiratory distress remains unclear. Current practice varies widely, with some clinicians opting for early intervention with the aim of preventing deterioration and others favouring a more expectant approach, with the aim of avoiding airway manipulation, potential complications, and costs associated with surfactant administration.

The lack of consensus about optimal management is partly due to the absence of high-quality evidence specific to this population. Existing studies have limitations in terms of sample size, study design, study population, and variability in clinical practices. This has resulted in a lack of clear guidance for clinicians, leading to inconsistencies in care.

The impact of respiratory illness in these infants extends beyond the neonatal period. Long-term respiratory morbidity, including impaired lung function and increased risk of respiratory infections, has been reported in individuals born both late preterm and early term [3, 5–8], and the underlying mechanisms and the role of early respiratory interventions in these long-term outcomes remain to be fully understood.

Given the significant clinical and economic burden associated with respiratory illness in late preterm and early term infants, there is a pressing need for evidence-based guidance on their management. A randomised controlled trial comparing early surfactant therapy to an expectant approach can provide valuable insights into the optimal timing and efficacy of this intervention. Such a trial would contribute to improving the care of these vulnerable infants and reducing the long-term consequences of respiratory disease.

### Objectives {7}

The trial will investigate whether, in late preterm and early term infants with respiratory distress, the early use of surfactant versus expectant management results in a shorter duration of hospital stay and fewer infants who fail to respond to treatment.

Primary objectives are as follows:

- To compare duration of neonatal hospital stay in infants randomised to receive early surfactant versus those who received expectant management.
- To compare incidence of severe respiratory failure in infants randomised to receive early surfactant therapy versus those who received expectant management.

Secondary objectives are as follows:

- To investigate the effects of early surfactant therapy versus expectant management on perinatal secondary outcomes.
- To investigate the cost-effectiveness of early surfactant therapy versus expectant management.

### Trial design {8}

This trial will be a pragmatic, multicentre, open-label, two-arm parallel-group, randomised controlled trial (RCT). An economic evaluation will be conducted alongside the clinical trial to assess the cost-effectiveness of early surfactant therapy versus expectant management.

A total of 1522 infants will be randomised to receive expectant management or early surfactant therapy. There will be a 12-month internal pilot phase, after which ‘stop-go’ criteria will be used to evaluate the feasibility of recruitment and other trial processes.

The trial data collection will be from trial entry until 1 year of age corrected for prematurity and will include screening, consent, treatment, and follow-up. Outcome information will be collected by case report forms (CRFs), with clinical data collection from medical records at the hospital. Questionnaires will be given to the mother at trial entry and around the time the infant is discharged from the hospital. Survival, paediatric secondary care use, and associated costs from discharge up to 1 year of age, corrected for prematurity, will be collected from a routine national database (e.g. HES), with no direct contact with the parent(s).



## Methods: participants, interventions and outcomes

### Study setting {9}

#### Study settings

The trial will be conducted across 45 neonatal units (NNUs) in the UK. Participating centres will include neonatal intensive care units (NICUs), local neonatal units (LNUs), and special care units (SCUs). For a full list of recruiting sites, please see Table 1: List of recruiting sites.

**Table 1:** List of recruiting sites

NHS trust	Recruiting site (hospital)
Bedfordshire Hospitals NHS Foundation Trust	Luton & Dunstable University Hospital
Belfast Health and Social Care Trust	Royal Jubilee Maternity Hospital, Belfast
Betsi Cadwaladr University Health Board	Glan Clwyd District General Hospital
Betsi Cadwaladr University Health Board	Wrexham Maelor Hospital
Betsi Cadwaladr University Health Board	Ysbyty Gwynedd District General Hospital, Bangor
Birmingham Women’s and Children’s NHS Foundation Trust	Birmingham Women’s Hospital
Bolton NHS Foundation Trust	Royal Bolton Hospital
Countess of Chester NHS Foundation Trust	Countess of Chester Hospital
Dartford and Gravesham NHS Trust	Darent Valley Hospital, Dartford
East Kent Hospitals University NHS Foundation Trust	William Harvey Hospital, Ashford
East Suffolk and North Essex NHS FT	Ipswich Hospital
Epsom and St Helier University Hospitals NHS Trust	St Helier Hospital, Carshalton
Great Western Hospitals NHS Foundation Trust	Great Western Hospital, Swindon
Hull University Teaching Hospitals NHS Trust	Hull Royal Infirmary
Imperial College Healthcare NHS Trust	Queen Charlotte's & Chelsea Hospital
Imperial College Healthcare NHS Trust	St Mary's Hospital, London
Isle of Wight NHS Trust	St Mary's Hospital, Isle of Wight
King's College Hospital, London	King's College Hospital, London
Lancashire Teaching Hospitals NHS Foundation Trust	Royal Preston Hospital
Leeds Teaching Hospitals NHS Trust	Leeds General Infirmary
Liverpool Women's NHS Foundation Trust	Liverpool Women's Hospital
Maidstone and Tunbridge Wells NHS Trust	Tunbridge Wells Hospital
Manchester University NHS Foundation Trust	Saint Mary's Hospital, Manchester
Manchester University NHS Foundation Trust	Wythenshawe Hospital, Manchester
Medway NHS Foundation Trust	Medway Maritime Hospital
Newcastle upon Tyne Hospitals NHS Foundation Trust	Royal Victoria Infirmary, Newcastle
NHS Lothian	Royal Infirmary of Edinburgh
NHS Tayside	Ninewells Hospital and Medical School, Dundee
Norfolk and Norwich University Hospitals NHS Trust	Norfolk and Norwich University Hospital
North Bristol NHS Trust	Southmead Hospital, Bristol
Northern Care Alliance NHS Foundation Trust	Royal Oldham Hospital
Northern Lincolnshire and Goole NHS FT	Diana, Princess of Wales, Grimsby
Portsmouth Hospitals University NHS Trust	Queen Alexandra Hospital, Portsmouth
Royal Devon and Exeter NHS Foundation Trust	Royal Devon and Exeter Hospital
Sheffield Teaching Hospitals NHS Foundation Trust	Jessop Wing, Sheffield
South Eastern Health and Social Care Trust	The Ulster Hospital, Belfast
South Tees Hospitals NHS Foundation Trust	James Cook University Hospital, Middlesbrough
South Tyneside and Sunderland NHS FT	Sunderland Royal Hospital
Southern Health and Social Care Trust	Craigavon Area Hospital
St George's University Hospitals NHS Foundation Trust	St George's Hospital, London
The Rotherham NHS Foundation Trust	Rotherham Hospital
The Shrewsbury and Telford Hospital NHS Trust	Princess Royal Hospital, Telford
University Hospitals Birmingham NHS FT	Birmingham Heartlands Hospital
University Hospitals Bristol and Weston NHS Foundation Trust	St Michael's Hospital, Bristol
University Hospitals Coventry and Warwickshire NHS Trust	University Hospital Coventry
University Hospitals Dorset NHS Trust	Poole Hospital
University Hospitals of Derby and Burton NHS FT	Queen's Hospital, Burton-on-Trent
University Hospitals of Derby and Burton NHS FT	Royal Derby Hospital
University Hospitals of Leicester NHS Trust	Leicester Royal Infirmary
West Hertfordshire Teaching Hospitals NHS Trust	Watford General Hospital

Forty-five participating NHS trusts and 50 recruiting hospital sites; the higher site count reflects that some trusts were recruited at 2 or 3 hospitals

### Eligibility criteria {10}

#### Inclusion criteria

- Born at 34^+0^ to 38^+6^ weeks of gestation
-  ≤ 24 h old
- Respiratory distress defined as follows:
  - *FiO*_*2*_ ≥ 0.3 and < 0.45 needed to maintain *SaO*_*2*_ ≥ 92%
  - *FiO*_*2*_ < 0.3 with clinically significant work of breathing
- Clinical decision to provide noninvasive respiratory support
- Written parental informed consent

#### Exclusion criteria

- Major structural or chromosomal abnormality
- No realistic prospect of survival
- Prior intubation and/or surfactant administration
- Known or suspected hypoxic ischaemic encephalopathy
- Congenital abnormality of the upper or lower respiratory tract
- Known or suspected neuromuscular disorder

## Participants

The trial population is late preterm infants (born at 34^+0^ to 36^+6^ weeks of gestation) and early term infants (born at 37^+0^ to 38^+6^ weeks of gestation) with respiratory distress, where a clinical decision has been made to provide noninvasive respiratory support.

## Site selection and training

Sites are identified through a site selection feasibility process and agreed to by the PMG. At the time of randomisation, a delegated clinician or advanced/associate neonatal nurse practitioner (ANNP), as per local scope of practice and the site delegation log, confirms participant eligibility and completes randomisation. The intervention, surfactant, is a stock drug for all recruiting sites and is administered and accounted for according to local policy and practice.

Selection criteria include the mandatory requirement for PIs to be suitably qualified, possessing current *Good Clinical Practice (GCP)* certification and appropriate research experience. Sites must demonstrate and *confirm adequate capacity and capability* (e.g. facilities, specialist equipment, and sufficient staff and patient throughput) to conduct the study successfully. Furthermore, sites are strategically selected based on their potential to maximize the *generalisability* of study results, which is achieved by ensuring inclusive recruitment across diverse populations, including those from areas of high deprivation or ethnic minority groups.

### Who will take informed consent? {26a}

Clinicians, advanced neonatal nurse practitioners, and nurses can obtain consent. They must have GCP, SurfON study training, and be delegated to take consent by the PI.

### Additional consent provisions for collection and use of participant data and biological specimens {26b}

Not applicable as study design does not involve the collection of biological specimens from participants. There is no plan to use the participant data for future, unspecified research. Long-term consent was obtained to contact participants in the future about further related research.

## Interventions

### Explanation for the choice of comparators {6b}

#### Rationale for expectant management group

Infants in the expectant management group will, where possible, be maintained on noninvasive respiratory support alone, at least until a more severe disease threshold is reached, defined as follows:

- Sustained (≥ 30 min) requirement for *FiO*_*2*_ ≥ 0.45 to maintain oxygen saturations (SaO_2_) ≥ 92%

The expectant management group serves as a control group to assess the natural course of respiratory disease in infants receiving only standard noninvasive respiratory support. This allows for the evaluation of the effectiveness of early surfactant therapy.

#### Rationale for early surfactant therapy group

Infants in the early surfactant therapy group will receive a single dose of surfactant administered as soon as possible after the infant has been randomised.

The early surfactant therapy group is designed to assess the potential benefits of early surfactant administration in improving outcomes for babies, mothers, and the NHS. This approach is based on the hypothesis that early intervention can prevent or limit deterioration in the respiratory condition and minimise the need for invasive respiratory support and mechanical ventilation, thus reducing the length of hospital stay for the baby and mother and associated costs.

#### Additional respiratory interventions

Information regarding the reasons for additional respiratory interventions and surfactant administration will be documented.

It is important to note that the decision to administer surfactant outside of surfactant administered according to allocation in either group will be based on the individual infant’s clinical condition and the attending clinician’s assessment of the risk-benefit ratio.

### Intervention description {11a}

The investigational medicinal product (IMP) is CUROSURF^®^, a natural animal-derived surfactant manufactured and supplied by Chiesi Farmaceutici S.p.A. at 96, Via San Leonardo, 43122 Parma, Italy. The Reference Safety Information (RSI) will be the latest approved Summary of Product Characteristics (SmPC) for the surfactant used.

The recommended starting dose for the IMP is 100–200 mg/kg (1.25–2.5 ml/kg), administered in a single dose as soon as possible after diagnosing respiratory distress. The administration of the IMP will be as per local site policy and procedure and may be completed by any clinician for whom this falls within their usual scope of practice, following confirmation of eligibility by a doctor or advanced neonatal nurse practitioner. As part of the intervention in this trial, surfactant will be given to infants as soon as possible after randomisation.

The IMP is a commonly used drug in NNUs and is being used in line with standard care. Side effects are uncommon and usually transient during administration.

The timing and dosage of IMP administration will be collected. Surfactant is a stock drug for all NNUs and will be administered and accounted for as per local practice. No specific contraindications are known.

### Criteria for discontinuing or modifying allocated interventions {11b}

Infants in the expectant management group should, where possible, be maintained on noninvasive respiratory support alone, at least until a more severe disease threshold is reached, defined as follows:

- Sustained (≥ 30 min) requirement for *FiO*_*2*_ ≥ 0.45 to maintain oxygen saturations (SaO_2_) ≥ 92%.

In either study group, surfactant may be given if intubation is required for another reason or if the attending clinician deems this necessary. Information on the reasons for additional respiratory intervention and surfactant administration will be collected.

### Strategies to improve adherence to interventions {11c}

Non-adherence to the trial intervention, particularly within the early surfactant therapy group, is closely monitored throughout the trial. The Data Monitoring Committee (DMC) has established specific criteria for monitoring non-adherence, defining it as instances where infants in the early surfactant arm do not receive the trial intervention, documented solely as ‘clinician decision’ without further explanation, and occurring more than once at a site.

The Trial Management Team will proactively review site data for these instances. Sites meeting the DMC’s criteria will be contacted by the trial management team to investigate the reasons for non-adherence and implement targeted interventions to reinforce protocol compliance. The DMC also closely monitors non-adherence cases as part of its ongoing safety and efficacy oversight, ensuring appropriate action is taken to maintain trial integrity. Furthermore, clear documentation requirements are emphasised to discourage ambiguous ‘clinician decision’ justifications, promoting transparency and accountability.

### Relevant concomitant care permitted or prohibited during the trial {11d}

There are no contraindicated medications or dietary supplements, and infants will be able to have all medicines and supplements normally prescribed for this population during the trial.

### Provisions for post-trial care {30}

Infants who develop health problems unrelated to the trial intervention will continue to receive all necessary clinical care through the NHS as part of routine practice. The trial does not restrict or limit access to ancillary care for conditions arising during the neonatal period.

The investigational product, CUROSURF^®^ (surfactant), is a licensed drug routinely used within the NHS for neonatal respiratory distress. Surfactant is administered only during the acute phase of illness and is not required once infants are discharged from hospital. Therefore, there are no specific posttrial care arrangements, and participants will continue to receive all clinically indicated treatments through the NHS.

In the event of harm to a participant due to someone’s negligence during the research, participants have grounds for legal action for compensation against the sponsor. The National Health Service complaint mechanisms will also be available to participants.

### Outcomes {12}

The primary and secondary outcome measures are detailed in Table 2.

**Table 2:** Primary and secondary outcome measures

**Outcome measures**	**Time point(s) of evaluation of this outcome measure**
•*Length of infant’s hospital stay* after birth, defined as the number of days from birth to discharge home from hospital	Between birth and discharge home from hospital
•*Incidence of severe respiratory failure*, defined as sustained (≥ 30 min) requirement for *FiO*_*2*_ ≥ 0.45 to maintain oxygen saturations (SaO_2_) ≥ 92%	Between trial entry and discharge home from hospital
**Perinatal clinical outcome measures**	**Time point(s) of evaluation of this outcome measure**
•*Total duration of NNU stay*, defined as total number of days of inpatient care in a neonatal unit (defined according to Healthcare Resource Group (HRG) Critical Care categories 1–5, 2016)	Between trial entry and infant discharge home from hospital
•*Total duration of neonatal intensive care*, defined as number of days of neonatal intensive care (defined according to HRG Critical Care categories 1–5, 2016)	
•*Duration of mechanical ventilation*, defined as days of ventilation via an ETT (endotracheal tube)	
•*Duration of noninvasive respiratory support*, using positive airway pressure or high flow	
•*Pulmonary air leaks*, requiring insertion of a chest drain	
•*Days of mother-infant separation*, defined using HRG Critical Care categories 1–5, 2016	
•*Breast milk feeding*, defined as (a) any breast milk feeding during neonatal hospital stay, (b) any breast milk feeding at hospital discharge, and (c) exclusive breast milk feeding at hospital discharge	
•*Late onset sepsis*, defined as the incidence of *microbiologically confirmed or clinically suspected* invasive infection more than 72 h after birth	
•*Need for inhaled nitric oxide (iNO) therapy*	
•*Need for extracorporeal membrane oxygenation (ECMO)*	
•*Medical respiratory diagnoses*, defined as any respiratory diagnosis attributed to the infant	
•*Surfactant administration*, defined as (a) administration of any additional surfactant in infants randomised to receive early surfactant or (b) administration of any surfactant in infants receiving expectant management, including number of doses and dose given	
•*Maternal length of hospitalisation*, defined as the total number of days spent by the mother in hospital after trial entry	Between trial entry and maternal discharge home from hospital
**Health economic outcome measures**	**Time point(s) of evaluation of this outcome measure**
•*Cost of maternal hospitalisation*	Between trial entry and infant discharge home from hospital
•*Self-reported maternal health-related quality of life*	
•*Costs associated with neonatal care*	
•*Survival, paediatric secondary care use, and associated costs* using routine national databases	Between infant discharge home and 1 year of age, corrected for prematurity

### Participant timeline {13}

Participant timeline: schedule of enrolment and interventions

**Table:** 

	**Trial period**				
	**Enrolment**		**Post-randomisation (after trial entry)**		**Close-out**
**Timepoint**	Within 24 h of birth		Immediately post-randomisation	At hospital discharge	At 1 year of age^a^
**Enrolment**					
Eligibility assessment	X				
Informed consent^c^		X			
Randomisation		X			
Intervention/comparator					
Intervention — surfactant administration^b^			X		
Comparator — expectant management (non-invasive respiratory support)			X		
Assessments					
Questionnaires (EQ-5D-5L; feeding)^c^			X	X	
Perinatal clinical data collection			X	X	
Follow-up data collection using routine national databases					X
Adverse events assessments (SAEs, SUSARs)			X	X	

^a^Corrected for prematurity

^b^Surfactant for infants randomised to early surfactant therapy will be administered as soon as possible after randomisation

^c^Provide questionnaire for completion only if the mother has provided consent to participate

### Sample size {14}

One of the primary outcomes is the length of hospital stay from trial entry to discharge home. Reported length of stay for late preterm infants with respiratory distress is estimated to be between 10 and 15 days from the literature [4, 10, 18]. Calculations are based on the reduction in median length of stay in NNU from 12 to 10 days in the early surfactant versus expectant management group. To detect a 2-day reduction in the length of stay (equivalent to a hazard ratio of 1.2) with 90% power and a two-sided 5% significance level, a total sample size of 1280 is required. We estimate the prevalence of multiple births in our patient population to be 25% [19] with an intra-class correlation coefficient of 0.9, based on previous perinatal trials. Allowing for an inflation factor of 1.13 for this and a 5% attrition rate, the required recruitment target is 1522 (761 per group). Based on data from NNUs, around 40–50 late preterm and early term infants per year require respiratory support. We aim to recruit an average of two infants per month over 30 months in approximately 45 NNUs to reach the target sample size of 1522, allowing for a staggered start.

The other primary outcome is the proportion of infants who reach a predefined higher threshold of disease severity, at which clinicians may choose to give ‘rescue’ surfactant. A recent study [20] reported an event rate of 45% in a cohort of late preterm infants admitted to neonatal intensive care for respiratory failure. This is likely to be lower in the trial population, which also includes early term infants. With a sample size of 1522, a treatment difference of 9% could be detected with 90% power and a two-sided 5% significance level if the expectant management group event rate was 45%. A smaller treatment difference could be detected if the expectant management group event rate (CER) was lower than this (e.g. 8% difference for a CER of 35%, 7% difference for a CER of 25%).

### Recruitment {15}

Given the estimated annual rate of late preterm and early term infants requiring respiratory support in participating NNUs, it is anticipated that an average of two infants per month can be recruited over a 30-month period. This will allow for a staggered start across the participating centres to reach the target sample size.

The SurfON trial aims to achieve adequate participant enrolment by implementing a multipronged recruitment strategy targeting potential participants (parents of eligible infants and pregnant women).

- Each site will maintain a screening log of all infants reviewed for eligibility, including those deemed ineligible and those whose parents decline participation. This systematic record allows monitoring of recruitment activity and identifies potential barriers to enrolment.
- Early engagement is an important component of the recruitment strategy. With the agreement of clinical teams, women expected to deliver between 34 and 36 weeks’ gestation may be screened for eligibility prior to birth. This enabled early identification of potential participants and maximised the available time to parents to consider participation. This approach helped to identify potential participants early and ensured that eligible infants were not missed due to time constraints immediately after delivery. In addition, clinicians were encouraged to approach parents of infants showing early signs of respiratory distress even before all eligibility criteria were fully met. This gave families additional time to consider the study and to make an informed decision should their infant’s condition progress to require noninvasive respiratory support.
- Awareness of the trial will be promoted using a variety of informational and recruitment materials. Posters, banners, and leaflets will be displayed in neonatal units to raise awareness among both staff and parents. These will be supplemented by other resources, including podcasts and parent-facing information leaflets, to support understanding of the trial and encourage participation. Together, these approaches aim to maximise trial visibility, ensure families are well informed, and facilitate sustained recruitment across all participating sites.

## Assignment of interventions: allocation

### Sequence generation {16a}

The allocation sequence will be generated by a bespoke system developed using the programming language PHP and database MySQL.

The randomisation programme will use a minimisation algorithm to ensure balance between the treatment groups with respect to week of gestation at birth, multiple birth, and centre. Infants will be randomised in a 1:1 allocation ratio to either the early surfactant therapy or expectant management group. Twins or higher-order multiple births will be randomised to the same arm.

### Concealment mechanism {16b}

A Senior Trials Programmer at the National Perinatal Epidemiology Unit (NPEU CTU) will write the randomisation programme and hold the treatment allocation codes. The randomisation programme will be hosted on a secure web-based platform. The IMP will be dispensed from the hospital stock through routine prescription. The senior trials programmer and trial statisticians will monitor the implementation of the randomisation procedure throughout the trial. Reports will be provided to the Data Monitoring Committee (DMC).

### Implementation {16c}

Randomisation of infants will be managed through a secure web-based randomisation facility hosted by the National Perinatal Epidemiology Unit Clinical Trials Unit (University of Oxford). Telephone backup will be available 24/7.

Randomisation will occur as soon as possible after eligibility is confirmed and informed consent is obtained. Staff at recruiting sites will log in to this system using a unique centre login to randomise participants. The system will prompt users to enter data to confirm eligibility, assign a unique SurfON trial identification (ID), and specify the randomised allocation for the infant.

For infants randomised to the early surfactant therapy group, surfactant administration should occur as soon as possible after randomisation.

## Assignment of interventions: blinding

### Who will be blinded {17a}

As SurfON is an open-label trial, participants and treating clinicians will not be blinded to treatment allocation. A validating statistician, who will remain blinded to treatment allocation, will independently check and confirm the primary comparative analyses after database lock. Full blinding of the trial statisticians is not possible, as interim reporting to the Data Monitoring Committee (DMC) requires an unblinded statistician to prepare safety and feasibility summaries. To limit potential bias, interim reports will be restricted to descriptive summaries only, without inferential comparisons between trial arms.

### Procedure for unblinding if needed {17b}

Not applicable as SurfON is not a blinded trial.

## Data collection and management

### Plans for assessment and collection of outcomes {18a}

Prior to any data collection, the mother must provide informed consent. Once consent is obtained, the randomisation process is initiated by filling out the appropriate forms on the SurfON randomisation website where the infant will be allocated *to early surfactant therapy* or *expectant management*.

Data for the SurfON study will be collected primarily on electronic case report forms (eCRFs) within the OpenClinica clinical database. Mandatory CRFs include the consent form (paper), randomisation, contact details, trial entry, trial intervention, respiratory support log, outcomes, and mother’s entry and discharge questionnaire (if consent is provided). Additional CRFs may be required for certain participants, such as the surfactant, hospital transfer, withdrawal, SurfON serious adverse event, and SurfON incident and deviation forms. These forms will be completed as needed.

Outcome data will be collected primarily from existing hospital records and electronic data collection systems. Data will be entered on trial-specific CRFs by site staff, with the outcome CRF completed at hospital discharge. Optional questionnaires may be administered to mothers who have consented, assessing quality of life and breast milk feeding. Maternal quality of life will be assessed using the preference-based EQ-5D-5L instrument. If an infant is transferred to another hospital, the recruiting site will remain responsible for data collection.

Secondary care use and survival data will be obtained from available electronic national databases in the UK. Databases will include information on hospital inpatient admissions, outpatient visits, paediatric critical care, and mortality data. This will include Hospital Episode Statistics (HES) in England and the electronic Data Research and Innovation Service (eDRIS) in Scotland. Parental consent will be obtained at trial entry to allow for follow-up using routine national databases.

### Plans to promote participant retention and complete follow-up {18b}

The Trial Co-ordinating Centre will maintain regular contact with all sites to ensure accuracy and completeness of all study documentation during the infants’ participation in the study. For infants who discontinue participation or where there is a deviation from protocol after the point of randomisation, sites will be asked to record reasons for this and to continue data collection for analysis of outcomes according to intention to treat.

Source data verification (SDV) is performed on the first 10 completed respiratory logs from each site, which record the primary outcome for the trial. Data are entered directly into OpenClinica, and paper copies are provided to the trial team for SDV checks. The trial team conducts manual SDV checks, provides individual feedback to sites, flags errors, and sends detailed emails highlighting common mistakes to prevent future errors. Additionally, the trial team hosts webinars, refresher training sessions, and drop-in sessions to address concerns and clarify proper completion of respiratory logs.

### Data management {19}

The Data Management Plan will outline the detailed procedures for data collection, cleaning, validation, and analysis to ensure the production of high-quality data for statistical analysis.

Source documents are where data are first recorded and from which infants’ CRF data are obtained. CRF entries will be considered source data if the CRF is the site of the original recording.

Authorised representatives from the sponsor, funder, research team, NHS trust, and regulators will have direct access to data for monitoring and auditing. Site staff will have restricted, password-protected access to the clinical database.

Clinical data will be entered into a validated database, with validation checks for accuracy. Participant names and identifiable information will be stored in a separate administrative database.

Electronic and paper records will be stored securely, with restricted access to authorised personnel. Electronic files will be stored on a secure server, with regular backups and restricted entry. Archiving of the Trial Master File will follow the requirements of the Clinical Trials Regulations, NPEU CTU Standard Operating Procedures, and applicable regulations, with a review of further archiving needs based on data protection laws. Data will be retained at recruiting sites in line with applicable NHS and trust policies.

At the end of the trial, all participant data will be transferred to the University of Leicester for long-term follow-up, in compliance with data sharing agreements.

### Confidentiality {27}

The trial will adhere to strict data privacy regulations, including the General Data Protection Regulation (GDPR) and Data Protection Act 2018. All documents will be stored securely at the NPEU CTU and accessible only to authorised trial staff. Personal identifiers and trial data will be stored in separate databases linked by the infant’s trial number. After the trial is completed and reports are published, data will be archived in a secure location with controlled access.

### Plans for collection, laboratory evaluation and storage of biological specimens for genetic or molecular analysis in this trial/future use {33}

Not applicable as there is no lab involvement.

## Statistical methods

### Statistical methods for primary and secondary outcomes {20a}

#### Primary outcomes

The statistical analysis will be conducted according to a pre-specified statistical analysis plan (SAP), which will be available in a separate document. The SAP will provide detailed information on the methods used to analyse the primary and secondary outcomes, including any adjustments for covariates and multiple comparisons.

Infants will be analysed in the groups to which they were randomly assigned, regardless of protocol deviations or actual treatment received (intention-to-treat (ITT) analysis population).

The primary analysis will be adjusted for minimisation factors (week of gestation at birth, multiple birth, and centre). The correlation in outcomes between multiple births will also be adjusted for, where technically possible.

For the length of hospital stay primary outcome, a time-to-event analysis using a Cox proportional hazards model will be conducted to calculate the hazard ratio and 95% confidence interval (CI). Kaplan–Meier curves will be plotted to visualise the survival functions. Median length of stay with interquartile ranges will be reported for each group. Deaths will be excluded (not censored) from the time-to-event analysis and reported separately.

For the severe respiratory failure primary outcome, risk ratios with 95% CI will be calculated using log-binomial regression or Poisson regression with a robust variance estimator in the event of non-convergence. Absolute risk difference and associated confidence intervals will also be calculated. 95% confidence intervals will be used for all comparative analyses, including subgroup analyses. *p*-values will be presented for the primary outcome comparisons with a two-sided 5% level of significance used for statistical inference. There will be no adjustment made for multiplicity when testing the two primary outcomes. Careful interpretation of the results will be based on the following: If both primary outcomes show evidence of superiority (i.e. evidence to reject the null hypotheses for both primary outcomes), this will be interpreted as providing robust evidence that early surfactant therapy is superior to expectant management. Similarly, if results show no evidence of a difference for both primary outcomes, this will be interpreted as providing no evidence that early surfactant therapy is superior to expectant management. In all other scenarios, the results will be interpreted cautiously and in the context of corroborating evidence from the secondary outcomes.

#### Secondary outcomes

For binary outcomes, risk ratios and 95% CIs will be estimated, following the same planned analysis as for the severe respiratory failure outcome. For normally distributed continuous outcomes, mean or median differences with 95% CIs will be calculated using linear regression. If the data show significant skewness, median differences with 95% CIs will be calculated using quantile regression. As per the primary outcomes, both crude and adjusted estimates will be presented, but the primary inference will be based on estimates adjusted for minimisation factors.

### Interim analyses {21b}

A Data Monitoring Committee (DMC), independent of the applicants and Trial Steering Committee (TSC), will review the progress and accumulating data from the trial at least annually and provide advice on the conduct of the trial to the TSC. DMC meetings will include open and closed sessions. The open session will cover recruitment, data quality, and pooled safety data. The closed session will focus on accumulating efficacy and safety data by trial arm. Outcomes will be reported with summary statistics, but treatment effects, *p*-values, and confidence intervals will be excluded unless requested by the DMC.

If appropriate, the DMC may recommend early stopping due, for example, to clear benefit or harm of a treatment or external evidence, e.g. published results of another trial. The TSC will review these recommendations and act upon them. If deemed appropriate, the TSC will carry the responsibility for deciding if the trial should stop recruitment early.

### Methods for additional analyses {20b}

#### Subgroup analyses

Pre-specified subgroup analyses will be conducted to examine the consistency of the effect of the intervention across gestational age groups (late preterm vs. early term) and first type of noninvasive respiratory support given (high flow vs. positive airway pressure) using the statistical test of interaction. 95% confidence intervals with a two-sided 5% level of significance for statistical inference will be presented.

No sensitivity analyses have been specified.

### Methods in analysis to handle protocol nonadherence and any statistical methods to handle missing data {20c}

Adherence to trial intervention will be reported by trial arm. Missing data will be described, for example, by presenting the number of individuals in the missing category. Missing data as a result of babies being lost to follow-up is expected to be minimal for short-term outcomes. No imputation of missing data is planned.

A restricted secondary analysis on the two primary outcomes will be performed, excluding infants who received surfactant outside of surfactant administered according to allocation without meeting the criteria for the severe disease threshold.

#### Economic evaluation

An economic evaluation will be conducted alongside the main clinical analysis. The statistical analysis of the economic evaluation will be conducted according to a pre-specified health economics analysis plan (HEAP), which will be available in a separate document. Therefore, only a brief account of health economic analysis is included in this protocol.

The primary objective of the health economic evaluation will be to calculate the 12-month cost-effectiveness of early surfactant therapy versus expectant management in a within-trial economic evaluation. This analysis will take the form of a cost-consequence analysis of key clinical primary and secondary outcomes alongside maternal quality of life, mother’s health care costs up to hospital discharge, and infant health care costs at 12-month follow-up.

A UK NHS perspective only including direct healthcare costs will be adopted for the analysis. Resource use will be collected for two different time periods: up to initial hospital discharge and from hospital discharge to 12-month follow-up. Initial hospital resource use will be collected both for babies and mothers (length of stay only). For infants, we will collect data on the inpatient days of high-, medium-, and low-level care needed after delivery and transfers out of the hospital. After initial hospital discharge, information on infants’ use of inpatient hospital stays, critical care whilst admitted, visits to accident and emergency (A&E) department, and hospital outpatient consultations will be collected up to the 12-month follow-up. All resource use will be valued in monetary terms using appropriate UK unit costs relevant to the time of analysis.

Maternal quality-adjusted life-years (QALYs) will be derived from utility scores obtained using the EQ-5D-5L quality-of-life instrument [21, 22]. For the cost-consequence analysis maternal QALYs, maternal health-related quality-of-life (HRQoL) domains and values from the EQ-5D-5L quality-of-life instrument will also be reported alongside the trial’s primary endpoints and selected key secondary outcomes. Measurements of maternal HRQoL using the EQ-5D-5L data will take place at baseline, initial hospital discharge, and 12-month follow-up.

The different components (costs and consequences) of the cost-consequence analysis will be presented in a disaggregated and tabulated manner and presented separately up to hospital discharge and at 1-year follow-up. Main outcomes for the cost-consequence analysis will be presented as mean estimates with associated 95% CIs for each group in the trial. Mean differences and associated 95% CIs between the two arms of the trial will be estimated using common statistical models including ordinary least squares or generalised linear models (GLM) regression analysis to adjust (if necessary) for potential imbalances at baseline.

### Plans to give access to the full protocol, participant level-data and statistical code {31c}

Prior to the recruitment of the first participant, the trial will have been registered on a publicly accessible database. The trial information will be kept up to date during the trial, and the chief investigator or their delegate will upload results to all those public registries within 12 months of the end of the trial declaration. All data requests should be submitted to the corresponding author of the main results paper for consideration. Access to de-identified patient data may be granted for secondary analysis following review. If approved, a data sharing agreement will be put in place. Data availability will begin with the publication of the final results.

## Oversight and monitoring

### Composition of the coordinating centre and trial steering committee {5d}

The trial will be overseen by a TSC, DMC, and a Project Management Group (PMG).

The Project Management Group, consisting of the CI and members of the NPEU Clinical Trials Unit, will conduct central monitoring to ensure the trial is conducted according to the protocol. The PMG will meet every 3 to 4 weeks. Central monitoring will include reviewing recruitment rates, data quality, and safety events.

The TSC will consist of independent members, including a chair, clinicians, statisticians, and patient and public involvement representatives. The TSC will meet at least annually to review the trial’s progress, provide scientific advice, and make decisions regarding the trial’s continuation or termination. The TSC will oversee the trial’s conduct, monitor safety and efficacy, and act upon recommendations from the DMC.

### Composition of the data monitoring committee and its role and reporting structure {21a}

The DMC will consist of independent members, including a chair, clinician, and statistician, who are separate from the trial team and TSC. During recruitment, the DMC will meet annually or more frequently as needed to review trial conduct, progress, and accumulating data. The DMC will then make recommendations to the TSC. The DMC Charter, outlining roles, responsibilities, and conduct, will be established at the initial meeting.

### Adverse event reporting and harms {22}

The safety reporting window for this trial will be from randomisation to infant’s discharge home.

An independent DMC will review study data, including serious adverse events (SAEs). The DMC is responsible for safeguarding participants’ well-being and will make recommendations to the TSC regarding trial continuation or protocol modifications. The TSC ultimately decides whether to terminate the trial for safety reasons.

On-site medical staff assess the relationship between adverse events (AE) and the trial supplement. However, due to the inherent characteristics of the patient population, determining causality can be challenging. Therefore, only unforeseeable serious adverse events will be documented under the trial’s safety reporting procedures.

Suspected unexpected serious adverse reactions (SUSARs) must be reported to the MHRA (Medicines & Healthcare Products Regulatory Agency) and the approving REC within 7 days if the event results in death or is life-threatening and within 15 days for all other SUSARs. Additionally, a copy of the SAE form related to the event will be forwarded to the Chair of the DMC for review.

Events of particular interest (which must also be reported as SAEs) are as follows:

- Death
- Transfer to another hospital related to early respiratory management
  ◦ For escalation of care
  ◦ For neonatal intensive care because of lack of intensive care cot capacity in the NICU or LNU of birth (NB, transfers for a lower level of care (SCU) or for reasons unrelated to respiratory management do not need to be reported as an SAE)
- Serious complication of ETT intubation such as hypoxia resulting in encephalopathy
- Severe pulmonary haemorrhage
- Severe intracranial haemorrhage

The full protocol provided to sites specifies foreseeable SAEs within the trial population that do not require reporting.

### Frequency and plans for auditing trial conduct {23}

The monitoring process will be independent of investigators and the sponsor.

The principal investigator (PI) will oversee the trial at their site, including recruitment, staff training, and ensuring data quality.

The NPEU Clinical Trials Unit (CTU) will develop a central monitoring plan based on the risk assessment. Recruitment patterns and data will be closely monitored. Any unexpected findings or outliers will be investigated, potentially leading to targeted site monitoring. Routine monitoring or auditing will not be conducted unless necessary, as determined by central monitoring.

### Plans for communicating important protocol amendments to relevant parties (e.g. trial participants and ethical committees) {25}

The NPEU CTU will submit and, where necessary, obtain approval from the sponsor, REC (Research Ethics Committee), and HRA (Health Research Authority) for all substantial amendments to the approved documents.

### Dissemination plans {31a}

The SurfON Collaborative Group intends to publish the protocol and peer-reviewed articles analysing key outcomes. All published material will acknowledge funding as required by the NIHR HTA.

The SurfON Collaborative Group will present data at national and international conferences and publish three open-access peer-reviewed articles. The NPEU Clinical Trials Unit will disseminate results at conferences and coordinate press releases, website promotion, and social media engagement. Parents will receive an email copy of the trial results, and the results will be disseminated through the trial website.

## Discussion

The SurfON trial is set to significantly influence clinical practice by providing essential evidence on the optimal timing for surfactant therapy in late preterm and early term infants with respiratory distress. By comparing early surfactant administration with expectant management, this study aims to resolve the current lack of consensus and substantial variability in treatment approaches. The findings will shape evidence-based guidelines, potentially enhancing respiratory outcomes and reducing healthcare resource use. Additionally, the results will lay the groundwork for future research into long-term respiratory health in this population and support the creation of standardised protocols for managing this common neonatal condition.

The COVID-19 pandemic presented significant challenges to trial conduct. Recruitment, initially planned for December 2019, was delayed until September 2020 due to pandemic-related restrictions. The subsequent 12-month pilot phase was severely impacted, achieving less than 50% of the targeted recruitment. This necessitated a pause in recruitment to address operational issues.

During this pause, intensive site communication and training activities were undertaken, along with the opening of additional sites. Following the resumption of recruitment, a substantial increase in monthly recruitment was observed. To compensate for the initial delays and achieve the target sample size of 1522, a 26-month extension was granted, setting a new end date of August 31, 2025. These experiences highlight the importance of adaptability and robust contingency planning in the face of unforeseen global events.

## Trial status

Protocol Version 7.0 and dated 25th April 2024. SurfON participant recruitment is still underway. Recruitment began in September 2020 and will continue until 30th April 2025.

## Data Availability

Data requests should be directed to the corresponding author for review and consideration. Please note that exclusive access to the data will be maintained until the publication of the main study findings. Subsequently, access to anonymised data may be granted upon a detailed review process.

## Abbreviations

AE: Adverse event
ANNP: Advanced neonatal nurse practitioners
AR: Adverse reaction
CI: Confidence interval
CRF: Case report form
CTU: Clinical Trials Unit
DMC: Data Monitoring Committee
ECMO: Extracorporeal membrane oxygenation
eDRIS: Electronic Data Research and Innovation Service
ETT: Endotracheal tube
HES: Hospital Episode Statistics
HRA: Health Research Authority
HRG: Healthcare Resource Group
HTA: Health technology assessment
IMP: Investigational medicinal product
iNO: Inhaled nitric oxide
MHRA: Medicines and Healthcare products Regulatory Agency
NHS: National Health Service
NIHR: National Institute for Health and Care Research
NNU: Neonatal unit
NPEU CTU: National Perinatal Epidemiology Unit Clinical Trials Unit
PI: Principal investigator
PMG: Project Management Group
SAE: Serious adverse event
